# Primary care at Swiss universities - current state and perspective

**DOI:** 10.1186/1756-0500-7-308

**Published:** 2014-05-22

**Authors:** Ryan Tandjung, Catherine Ritter, Dagmar M Haller, Peter Tschudi, Mireille Schaufelberger, Thomas Bischoff, Lilli Herzig, Thomas Rosemann, Johanna Sommer

**Affiliations:** 1 Institute for Primary Care and Health Services Research, University of Zurich, Zurich, Switzerland; 2 Primary Care Unit, University of Geneva, Geneva, Switzerland; 3 Institute of General Practice, University of Basel, Basel, Switzerland; 4 Bernese Institute of General Practice, University of Bern, Bern, Switzerland; 5 Institute of General Practice, University of Lausanne, Lausanne, Switzerland

**Keywords:** Primary care, Swiss universities, Research output, Teaching, Academic career, Medical education

## Abstract

**Background:**

There is increasing evidence that a strong primary care is a cornerstone of an efficient health care system. But Switzerland is facing a shortage of primary care physicians (PCPs). This pushed the Federal Council of Switzerland to introduce a multifaceted political programme to strengthen the position of primary care, including its academic role. The aim of this paper is to provide a comprehensive overview of the situation of academic primary care at the five Swiss universities by the end of year 2012.

**Results:**

Although primary care teaching activities have a long tradition at the five Swiss universities with activities starting in the beginning of the 1980ies; the academic institutes of primary care were only established in recent years (2005 – 2009). Only one of them has an established chair. Human and financial resources vary substantially. At all universities a broad variety of courses and lectures are offered, including teaching in private primary care practices with 1331 PCPs involved. Regarding research, differences among the institutes are tremendous, mainly caused by entirely different human resources and skills.

**Conclusion:**

So far, the activities of the existing institutes at the Swiss Universities are mainly focused on teaching. However, for a complete academic institutionalization as well as an increased acceptance and attractiveness, more research activities are needed. In addition to an adequate basic funding of research positions, competitive research grants have to be created to establish a specialty-specific research culture.

## Background

There is increasing evidence that a strong primary care is crucial for an efficient health care system [1-3]. A strong primary care does not only provide better health outcomes with lower cost, it also favors equal access to healthcare for the entire population. We based our definition of primary care on the one provided by the European section of the World Organisation of Primary Care (WONCA) [4]. Primary care is the first contact in healthcare for many patients and has therefore an important role in the coordination of care [5]. Demographic changes and an increasing number of patients with chronic diseases and multimorbidity will not only increase the need for primary care physicians (PCPs), but also for research on how to organize primary (and secondary) care in the future [6]. In contrast, Switzerland is facing a shortage of PCPs. Studies indicate that primary care is not attractive as a career option [7-9]. A survey at the medical school of Basel in the year 2005 showed that at the end of medical school only 10% of medical students were interested in a career in primary care [9]. In a cohort of young doctors in their third year of residency in the year 2001, only 9.7% aimed to become PCPs [8]. One reason for this is probably that primary care is not sufficiently present as an academic field within universities. As a consequence, universities and politicians became aware that the role of academic primary care should be strengthened; this led to the funding of institutes of primary care at all Swiss medical faculties (Basel, Bern, Geneva, Lausanne and Zurich). Switzerland has five medical faculties/medical schools and each of them are associated with the local university. The structure of an institute is comparable to that of a department at universities in other countries.

The Federal Council of Switzerland has recently initiated the “master plan for primary care” [10] a multifaceted political intervention program to strengthen the role of primary care and to prevent a further shortage of PCPs. The master plan also aims to strengthen the academic presence and research efforts in primary care. In this paper we present a baseline assessment of the teaching and research activities at the Swiss institutes of primary care.

## Methods

### Design

#### Cross sectional survey

##### Sample

Five institutes for primary care in Switzerland located at the universities of Basel, Bern, Geneva, Lausanne and Zurich.

### Measures

The institute in Geneva developed a questionnaire based on literature review. The questionnaire was sent to the head of each of the five institutes. It collected information about human resources in full-time-equivalent (FTE), allocated to the domains of research, administration and teaching. The inventory included PCPs in private practice teaching medical students (courses, one-to-one-tutorial). Furthermore financial resources, organisational integration, the role of the institute in teaching (involvement in lectures, courses, one-to-one-tutorials) and research (human resources, main research projects, publications) were assessed. Publications were divided into main authorship (first or last author) and co-authorship publications of members of the institutes and into peer-reviewed and non-peer-reviewed articles. A publication was only counted once, regardless of the number of authors involved. Data on publications and third party funding was collected for the time period between 2008 and 2012. We chose the same time period for all institutes to allow a comparison. Data on teaching hours and involved PCPs in teaching courses were based on the academic year from autumn 2011 to summer 2012. Data collection took place between October 2012 and January 2013. Stated human resources reflected the situation in December 2012.

In a second step, a member of the primary care unit of Geneva visited the institutes and an interview was conducted with the head of each institute for more detailed information about stated domains and to clarify definitions and answers provided in the written questionnaire.

### Analysis

First we compared all institutes in the domains of human and financial resources, research funding and research output and teaching. In a second step we specifically compared the institutes with the highest output in research in the dimension of research funding, human resources and teaching load to the other institutes.

### Ethical approval

This study collected data on academic infrastructure, research and teaching activities of academic staff. No information about patients was collected. Therefore, according to the current law in Switzerland this study does not need ethical approval. All heads of the institutes provided informed consent to a non-anonymized publication of the data for each institute.

## Results

### Main findings

**Figure 1:**
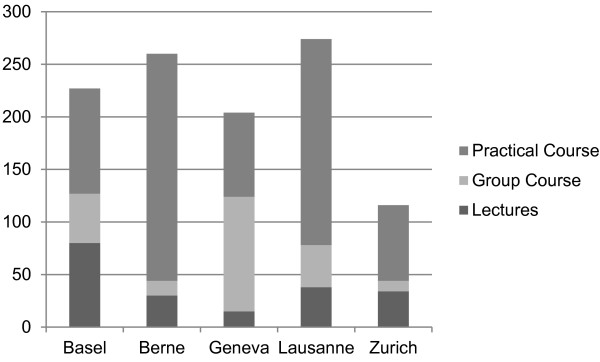
**Presence of primary care in the five medical faculties, the figures in the y-axis indicated total hours for each medical student.** All hours of the three teaching formats: lectures, group courses (university based) and practical courses (based in primary care practices) are listed to a total addition over the six years of undergraduate training. Additional courses (special interest courses) are not counted.

In the following sections the results are presented by each university, starting alphabetically. The teaching hours are presented from the perspective of a student and are only covering courses provided for all students. We did not include optional teaching content (e.g. special lectures or courses for a small number of students with particular interests in primary care). An overview of the main findings for all institutes is shown in Table 1. Figure 1 presents the total hours of primary care teaching format of a medical student at each university. We did not find a correlation between research output and research funding. However, the institute with the highest research output has the lowest teaching load and the highest established positions devoted to research.

**Table 1:** Overview of human resources, teaching and research figures of the five institutes for primary care in Switzerland

	**Basel**	**Bern**	**Geneva**	**Lausanne**	**Zurich**
**Established positions of each institute** (in full-time equivalent)					
Teaching	1.4	2.05	1.1	0.9	1.15
Research	0	0.7	0.4	0.4	4.0
Administration	0.5	2.4	0.4	0.8	1.1
Total	1.9	5.15	1.9	2.1	6.25
**Primary care physicians (PCPs) involved in teaching** (number of PCPs)					
Number of PCPs involved in teaching medical students	250	602	91	200	188
**Publications** (number of publications in the years 2008–2012)					
Peer reviewed articles	21	4	14	4	220
Non peer reviewed articles	74	11	17	9	146
Peer reviewed articles co-author	17	2	15	8	62
Non peer reviewed articles co-author	17	2	15	7	3
**Research funding** (in Swiss franc, 2008–2012)					
Research funding as investigators	1′500′000	350′000	347′280	240′000	2′511′074
Research funding in project as co-investigator	175′000	70′000	1′956′010	-	-
**Research fields**					
Main interests and research activities	Teaching in primary care, cardiovascular risk management	Teaching in primary care, infectious diseases, prevention	Communication, adolescent medicine, teaching in primary care	Diagnostic testing in primary care	Health services research, chronic care (mainly COPD and diabetes)

Table 1 Listed are the established positions for academic positions (research, teaching) for researchers and administrative personnel, figures represent full time equivalent (FTE). Positions financed by external sources or research grants are not listed. The number of primary care physicians involved in teaching courses (taking place in primary care practices and not at universities) is listed separately. Publications were counted in the time period between 2008 and 2012, publications with a team member as first or last author were counted as authorships; publications were only counted once per institute, regardless of the number of participating authors. Third party funds are reflecting the same time period (2008–2012) and are listed in Swiss franc (CHF).

### Basel

#### Organisation

The institute of general practice in Basel (Institut für Hausarztmedizin Basel, IHAMB) was founded in 2005; the institute is associated to the medical faculty. So far, there is no established chair.

#### Teaching

The medical school in Basel has a yearly capacity of 170 medical students. The IHAMB is present with different teaching formats (lectures, group courses, practical work and exams) in all six years of undergraduate training. During the undergraduate training a student in Basel has 80 hours of lectures held by PCPs, 47 hours of group courses, and around 100 hours of one-to-one tutorials. The IHAMB presents a broad spectrum of subjects (first aid, a variety of different clinical subjects, e.g. cardiovascular diseases). The one-to-one tutorial in Basel takes place in the first year of the master degree (4th year of undergraduate training) with a course length of 20–24 half-days. 250 PCPs are involved as tutors. The PCPs are paid 3000 Swiss francs (CHF) for the entire course, which corresponds to about 150 CHF per half-day. The total amount represents about 50% of the yearly budget of the IHAMB.

#### Research and publications

The institute has no established research position, research activities are paid by third party-funds. The institute participates in studies concerning cardiovascular risk assessment in primary care, teaching in primary care practices (evaluation of one-to-one tutorial) and in studies related to workforce issues in primary care [11,12]. The publications are mainly focused on shortage of PCPs and on the future of primary care (mainly non-peer-reviewed articles). The IHAMB is involved in the supervision of medical students writing their master thesis (approximately 6 master theses/year), or their medical dissertation (3–4 dissertations completed every year).

### Bern

#### Organisation

The Bernese institute of general practice (Berner Institut für Hausarztmedizin, BIHAM) was founded in 2009; it is affiliated to the medical faculty of the University of Bern. The BIHAM has PCPs with didactical background, but no PCP with a research background.

#### Teaching

The medical school in Bern has a yearly capacity of 180 medical students. PCPs in Bern have a long tradition in teaching and first started activities in 1983. The BIHAM is present with teaching contents in all six years. Over the six years of undergraduate training a student in Bern has 30 hours of lectures held by PCPs, 12 hours of group courses, and 216 hours spent in a primary care practice (one-to-one-tutorial). The lectures and group courses of PCPs cover broad clinical content of different clinical specialties and non-clinical subjects, such as communication skills, phone consultation. The one-to-one-tutorial takes place from the first to the fourth year of undergraduate training with the same PCP over all four years; the tutorials take place over 8 half-days each year of the bachelor studies and three weeks in the first year master degree (4th year), so overall 54 half-days. 602 PCPs are involved in teaching to medical students. The PCPs are globally paid: 1200 CHF for a bachelor student, 4500 CHF for a master student corresponding to a remuneration of 150 CHF per half-day. The total amount corresponds to 69% of the yearly budget of the institute.

#### Research

The institute has one established position of a research associate (0.7 FTE. A further position (0.5 FTE research associate) is paid through third-party funds. The institute participates in clinical research projects (urinary tract infections, consulting parents with obese children) and projects in the field of teaching [13,14]. On average 5 Master theses of medical students are supervised each year.

### Geneva

#### Organisation

The primary care unit of Geneva (unité de médecine de premier recours, UMPR) was founded in 2009 and is affiliated to the medical faculty of the University of Geneva. The unit is part of the primary care institute of the University of Geneva which has the same director as the medical policlinic of Geneva University Hospitals.

#### Teaching

The medical school of Geneva has a yearly capacity of 140 medical students. Teaching activities began in 1993 much before the unit was created. The unit is responsible for teaching primary care to medical students in the first three years (bachelor degree) and participates in teaching courses of the master studies, but is not responsible for this content (it is under the responsibility of the Primary Care division of Geneva University Hospitals). During the six years of undergraduate training, a medical student in Geneva has 15 h of lectures held by PCPs, 109 hours of group courses and 80 hours of practical courses in a primary care practice. The lectures are held during the first two years of undergraduate training; PCPs participate in teaching of group courses from the 2nd to the 5th year in subjects such as communication, clinical epidemiology, medical humanities and physical examination. Four half-days in the second and eight half-days in the fourth year (first year master degree) are spent in a primary care practice, in total 12 half-days. 91 PCPs participate as tutors in primary care. The remuneration is 145 CHF per half-day for a bachelor student and 290 CHF per half-day for a master student (overall 2380 CHF per tutor).

#### Research

A research position of 0.4 FTE is provided by the university. The main focuses are research projects about physician-patient-communication; adolescent medicine and about teaching and teaching formats in primary care [15,16]. The unit participates in the supervision of master-thesis of medical students; with an average of about 3 master-theses per year.

### Lausanne

#### Organisation

The institute of general practice in Lausanne (Institut Universitaire de Médecine Générale IUMG) was founded in 2007 and is part of the medical policlinic of the University hospital of Lausanne; the IUMG is affiliated to the faculty of biology and medicine of the University of Lausanne. There is no established chair.

#### Teaching

The medical school in Lausanne has a yearly capacity of 160 medical students. The IUMG is present with teaching contents over all six years. Within the six years of undergraduate training a medical student has overall 38 hours of lectures held by PCPs, 40 hours of group courses and 196 practical courses in primary care practice. The IUMG presents a variety of subjects (history taking, communication skills, and specific diseases e.g. dementia) in the first four years. Practical courses are present from the second to the fifth year. There is a mandatory full month at a PCPs office in the 5th year (practical year). 200 PCPs participate in teaching courses for medical students. Students spend one half-day in primary care practice in the second year, two days in the third and fourth year, as well as a month (42 half-days) in the fifth year; so overall 51 half-days. The remuneration is 360 CHF per half-day for bachelor students and in the first master year and 100 CHF per day (assuming one hour of teaching) for each day of the monthly internship (overall 2000 CHF). The total budget accounts for about a third (32%) of the yearly budget of the IUMG.

#### Research

The IUMG has a 0.4 FTE position for research, this position is paid by the university. Research projects relate to different clinical topics and diagnostic tests in primary care (thoracic pain, depression, somatoform diseases, diabetes, anaemia and fatigue, health literacy) [17,18]. The IUMG supervises on average 4 master theses of medical students per year.

### Zurich

#### Organisation

The institute for primary care Zurich (Institut für Hausarztmedizin Zürich, IHAMZ) was founded in 2008. The institute has an established chair and is an independent institution of the medical faculty of the University of Zurich and the University hospital of Zurich.

#### Teaching

The medical school in Zurich has a yearly capacity of 250 medical students. In six years of undergraduate training, a medical student in Zurich has 34 hours of lectures held by PCPs, 10 hours of group courses and 72 hours of practical work (36 hours in one-to-one-tutorials, 36 h in group courses with 2 students per PCP). Lectures are mainly focusing on the role of primary care in healthcare, prevention, and differential diagnosis in a low-prevalence setting. 188 PCPs participate in teaching courses for medical students (one-to-one-tutorial and group course in primary care practices for 2 students); the remuneration is 147 CHF per half-day.

#### Research

The institute has 4 FTE positions for research, paid by the university. The IHAMZ has the main focus in health services research. It conducts a variety of clinical studies in primary care (e.g. chronic care in diabetes, COPD; diagnosis of skin cancer) and in projects concerning electronic health records (**f**amily medicine **i**nternational classification in primary care **r**esearch using **e**lectronic medical records, FIRE) [19,20]. The IHAMZ supervises master-theses of medical students, on average 10 master theses of medical students were supervised, furthermore an average of 5 medical dissertations per year were completed every year.

## Discussion

The academic primary care research and teaching activities vary widely between the five Swiss universities. Overall, teaching has a longer tradition and higher financial resources. So far the institute in Zurich is the only one with a chair; but two other institutes (Bern, Basel) have announced they will establish a chair in primary care by 2014. The human resources show comparable resources within teaching, but very low resources in research with Zurich the only exception. Limited research resources are also reflected in the scientific output of the institutes.

### Teaching

The teaching activities of the five institutes cover broad clinical content, but their presence during the six years of undergraduate training is very inconsistent, Zurich and Geneva have a smaller role than the other institutes, when comparing total teaching hours in primary care, which is mainly due to the much shorter length of placements in primary care practices. A detailed comparison of the teaching activities is difficult, since undergraduate curricula at the five universities vary substantially within Switzerland. All medical faculties have the same learning objectives [21] and final examinations; and recently all conducted a curricular reform of undergraduate training, introducing the bachelor and master degrees (“Bologna model”). Nevertheless the structures of each medical faculty remain very different, e.g. in Bern and Geneva lectures are reduced to a minimum in order to focus on problem-based learning, whereas other medical faculties, e.g. Zurich, have a curriculum with a greater amount of lectures, [22,23] limiting the scope for comparison. In all universities PCPs are involved in lectures and tutorials in relation to communication and medical humanities. Yet, beyond the medical humanities there is no unifying subject, the variety of medical subjects range from prevention, cardiovascular diseases, palliative medicine, life cycles, first aid, to medical confidentiality. There is currently no clear curriculum defining what should be taught in primary care (chronic diseases and patient education such as in COPD, diabetes, asthma etc.; acute conditions seen in primary care, etc.). These findings underscore the need for PCPs to develop a clearer academic teaching agenda beyond medical humanities.

Early contact to PCPs and primary care has been shown to be effective in increasing interest for choosing primary care as a career [8,24-28]. Facing the shortage of PCPs, all medical faculties integrated more practical courses in primary care practices, as e.g. the one-to-one-tutorial first established in Basel [12]. Two medical faculties have longer and mandatory placements with three (Bern) and four (Lausanne) weeks spent in a primary care practice. Furthermore, Bern has a continuity of regular placements in primary care over four undergraduate years, spent with the same PCP. The other universities have no mandatory long-term placements in primary cares (Geneva, however, will have one similar to Lausanne as from 2015) and short-term placements are usually limited to 10 within 6–12 months. These shorter placements limit the exposure to characteristics of primary care such as continuity of care. Current research indicates that early and maximized contact with primary care increases the interest for a career as PCP [29-33]. This also implies a need for continuous presence of primary care through all six years of undergraduate training. A total of 1331 PCPs are involved in courses or tutorials throughout Switzerland, including some PCPs who are involved in teaching activities at more than one university. Compared to Germany with around 3700 PCPs involved in teaching activities in primary care [34], this is an impressive number since Germany’s population is more than ten-fold that of Switzerland. The remuneration for these courses is a substantial part of the budgets of the institutes and considerably higher than the average remuneration in Germany (25 Euros = 30 CHF/day) [34]. The comparison within the Swiss universities is difficult; since courses are organised very differently and payment schemes differ even within the same medical faculty. In the upcoming years the number of medical students in Switzerland will increase: a major challenge will therefore be to raise the numbers of teaching PCPs while in the meantime ensuring a high didactic quality, e.g. with regular didactic courses [35].

### Research

While teaching in primary care in all five medical faculties has been strengthened in the last decade the role of research remains very small, as in many other countries, e.g. in Eastern Europe [36]. All institutes have at least a small research activity, but only Zurich has enough resources resulting in a substantial amount of research projects and publications. Compared to Germany, where the development of academic primary care is slightly more advanced [34], the research output in Zurich is comparable to leading German institutes [37,38], even though a gap still exits compared to countries where academic primary care is much more developed (United Kingdom, The Netherlands) [39]. Furthermore the research output of the institute in Zurich is comparable to other medical specialties at the University of Zurich [40].

The institutes in Basel and Zurich all have substantial third party funding, compared to German institutes [34]. Basel and Zurich institutes rank in the top for the field as only two German institutes managed to raise more (with an advantage of 5–7 years) with third party funds of 3.5-4 Mio Euros (4.2-5 Mio. CHF). However the comparison of absolute numbers is limited - especially considering the higher wages in Switzerland. Interestingly, our survey did not show a clear correlation between third party funding and research output, emphasizing that PCPs with appropriate research skills are at least as important as financial resources. However, the institute with the highest research output has also the lowest teaching load. A possible reason might be that the different tasks in teaching or research also attract PCPs with different backgrounds and interests. This aspect should inform recruitment of future staff at the institutes.

Nevertheless established resources – with the exception of the institutes in Zurich – are very small and represent therefore a major limitation to expanding research activities, which clearly is reflected in the comparison of research output. There is increasing evidence that a strong primary care is essential for an efficient health care system, but more research has to be conducted [1-3,6]. In primary care research Switzerland lags far behind leading countries such as the United States, UK and The Netherlands [39]. Primary care research can also contribute to increasing the attractiveness of the field. The low attractiveness of primary care for medical students and residents in Switzerland is known [8,41]; especially in male students, where prestige and research often play a more important role [26,42,43]. The implementation of research in primary care opens more career opportunities for future PCPs and might therefore make a career as a PCP more attractive [44]. The next most important step is to establish resources specifically allocated to research positions, which will also enable the institutes to raise more third party funds.

### Strengths and limitations

The academic institutes of primary care in Switzerland are young and this is the first inventory regarding teaching and research activities. The institutes are usually affiliated to both, the local university and the local university hospital; these very different structures might sometimes limit a direct comparison of our results. A limitation of our study is that we used simple metrics to measure teaching and research outputs: teaching activities were measured as number of hours and research output as number of publications. We could not retrieve cumulative impact factor or H-indices [45]. This limits the comparison with the international research field. However we think our inventory of the five institutes provides an indication of where we stand internationally and highlights the need for more investments in academic primary care in our country.

## Conclusion

So far, the academic activities of the five institutes for primary care at Swiss Universities are mainly focused on teaching. Despite this focus a clear ongoing primary care curriculum throughout the years is missing. This curriculum should include more clinical and academic topics relevant to primary care (such as chronic and acute conditions seen in primary care) in addition to medical humanities. It should also include more time spent in the general practice. For a complete academic institutionalization as well as an increased acceptance and attractiveness, more research activities are needed. In addition to an adequate basic funding of research positions, competitive research grants need to be created to establish a specialty specific research culture.

## Competing interests

All Authors declare no conflict of interest. All authors are staff members of one of the five institutes and were financed by these institutions.

## Authors’ contributions

RT was involved in data analysis and was responsible for the first draft and final version of the manuscript. CR contributed to the conceptual work of the questionnaire, collected data and conducted the interviews; DH contributed to the conceptual work of the questionnaire and was involved in data analysis. PT, MS, TB, LH contributed to the data collection and analysis. TR contributed substantially to the analysis and drafting of the manuscript. JS had substantial contribution in conceptualisation of the questionnaire, data analysis and manuscript. All authors have given the final approval of this final version.
